# SNAFU: The Semantic Network and Fluency Utility

**DOI:** 10.3758/s13428-019-01343-w

**Published:** 2020-03-03

**Authors:** Jeffrey C. Zemla, Kesong Cao, Kimberly D. Mueller, Joseph L. Austerweil

**Affiliations:** 1 Department of Psychology, University of Wisconsin-Madison, 1202 West Johnson Street, Madison, WI 53706 USA; 2 Department of Computer Sciences, University of Wisconsin-Madison, 1210 West Dayton Street, Madison, WI 53706 USA; 3 Department of Communication Sciences and Disorders, Wisconsin Alzheimer’s Institute, and Wisconsin Alzheimer’s Disease Research Center, University of Wisconsin-Madison, 1975 Willow Drive, Madison, WI 53706 USA

**Keywords:** Verbal fluency, Semantic networks, Memory retrieval, Methodology

## Abstract

The verbal fluency task—listing words from a category or words that begin with a specific letter—is a common experimental paradigm that is used to diagnose memory impairments and to understand how we store and retrieve knowledge. Data from the verbal fluency task are analyzed in many different ways, often requiring manual coding that is time intensive and error-prone. Researchers have also used fluency data from groups or individuals to estimate semantic networks—latent representations of semantic memory that describe the relations between concepts—that further our understanding of how knowledge is encoded. However computational methods used to estimate networks are not standardized and can be difficult to implement, which has hindered widespread adoption. We present SNAFU: the Semantic Network and Fluency Utility, a tool for estimating networks from fluency data and automatizing traditional fluency analyses, including counting cluster switches and cluster sizes, intrusions, perseverations, and word frequencies. In this manuscript, we provide a primer on using the tool, illustrate its application by creating a semantic network for foods, and validate the tool by comparing results to trained human coders using multiple datasets.

## Introduction

People store and retrieve knowledge with relative ease. The way that we represent knowledge in the mind and the mechanisms that allow efficient retrieval have long been a topic of scientific interest (Quillian, 1967; Collins & Quillian, 1969; Tulving, 1972). One method for examining how memory retrieval operates is to analyze how people respond to a simple memory probe. The verbal fluency task (Bousfield & Sedgewick, 1944) is a popular experimental paradigm in which participants are provided a category probe and asked to list as many items from that category as they can in a fixed period of time (typically 1–3 min). There are two common variants of the verbal fluency task: In the *semantic fluency task*, participants list items from a fixed semantic category (e.g., animals), while in the *letter fluency task*, participants list items that begin with a particular letter (e.g., words that start with the letter F).

The task is used broadly in many areas of psychology, including the study of bilingualism (Gollan et al., 2002), aging (Hills et al., 2013), and clinical disorders such as Alzheimer’s disease (Monsch et al., 1992). The fluency task is also included in many popular neuropsychological batteries, such as the Cognitive Linguistic Quick Test (Helm-Estabrooks, 2001) and Montreal Cognitive Assessment (Nasreddine et al., 2005).

Fluency data are richly structured: the number of items recalled and ordering of items follow predictable patterns. For example, in the semantic fluency task, responses from healthy individuals group into semantically related clusters (e.g., listing *lion, giraffe,* and *hippo* together because they are all African animals; Troyer et al., (1997)). One issue with the analysis of fluency data is that scoring the data can be subjective. Clusters can be defined along a number of dimensions—such as geographical (e.g., African), taxonomic (e.g., dogs), relational (e.g., cats and mice), or perceptual (e.g., large animals). There is significant variability in the literature over what counts as a valid response (Jordan, 2014). For example, it is at the discretion of the researcher whether or not to allow fictional animals (e.g., *dragons*), farm and husbandry terms (e.g., *steed*), or age-dependent terms (e.g., *calf* ). What counts as a valid response, and what are the appropriate cluster labels to use? These choices lead to differences across research groups and can have an impact on the scientific conclusions (Abwender et al., 2001; Ross et al., 2007). The lack of standardization makes scoring the fluency task prone to idiosyncrasies and researcher degrees of freedom (Simmons et al., 2011), which have been implicated as possible culprits for the recent “replication crisis” (Open Science Collaboration et al. 2015).

Hand-coding clusters in fluency data is time consuming, which has recently led to the development of statistical approaches for identifying clusters (e.g., Kim et al., (2019), Linz et al., (2017), and Woods et al., (2016)). For instance, Linz et al., (2017) use a distributional semantics model trained using word2vec (Mikolov et al., 2013) to estimate animal similarity and demarcate clusters. While these approaches have been successfully validated on a few datasets, they may not capture all of the way humans mentally categorize concepts. While statistical cluster scoring techniques will likely continue to improve, the vast majority of research using verbal fluency tasks continue to rely on hand-coded clustering.

The rich structure of fluency data stems from the mental organization of semantic concepts and the retrieval processes used to recall them (Hills et al., 2012; Abbott et al., 2015). Computational methods have been developed to estimate semantic networks (abstract representations of semantic memory) from fluency data that reveal this structure. However, these methods can be difficult and time consuming to implement. As a result, a semantic network analysis of fluency data is rarely performed. Some network estimation methods are worse than others at capturing human behavior (Zemla and Austerweil, 2018), but choosing an estimation method is still ad hoc and often based on ease of implementation. Further, not having standards and best practices can lead to the temptation of selecting a network estimation method based on which one provides the desired results (as well as more innocuous forms of motivated data analysis). As such, network analysis of fluency data is still relatively uncommon and the reliability of many analyses is mostly unknown.

In this article, we present SNAFU: the Semantic Network and Fluency Utility. SNAFU is a tool for analyzing fluency data that aims to increase transparency, reproducibility, and interpretability of verbal fluency analyses. SNAFU automates many common approaches to quantifying fluency data, including computing cluster sizes and switches, word frequencies, age-of-acquisition, intrusions, and perseverations. SNAFU also implements a number of methods for estimating networks from fluency data, and uses current best practices as defaults (Zemla & Austerweil, 2018). SNAFU comes in two flavors: (1) a Python library for programmatically analyzing fluency data, and (2) a graphical user interface (GUI) that provides an easy point-and-click interface for analyzing fluency data. The Python library is cross-platform and built and tested using Python 3.5. The GUI is available for download on Windows and macOS.

## Python library

The Python library contains a set of tools for analyzing fluency data. It provides more flexibility than the GUI, but is intended primarily for researchers who have some programming experience. The library is open-source and available for download on GitHub at https://github.com/AusterweilLab/snafu-py. For convenience, it can also be downloaded from the command line using pip^1^ (see Code Snippet 1).

**Code Snippet 1:**
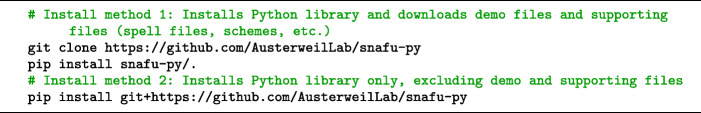
SNAFU can be installed via pip

A large semantic fluency dataset spanning several categories (animals, fruits, vegetables, foods, supermarket items, and tools) is included on the GitHub repository. The repository also includes a demo file with all of the code snippets in this manuscript (*brm_demo.py*) and several additional demo files covering various use cases. The following sections provide a high-level overview for how to analyze your fluency data with SNAFU.

### Formatting and loading a dataset

SNAFU requires that a data file is formatted as a comma-separated value (CSV) file with a header row. The GitHub repository includes a sample dataset of semantic fluency combined from three experiments (collected between 2015 and 2017), containing 807 lists from 82 participants, with a total of 24,572 responses. To load data into SNAFU, the data file must contain a minimum of three columns designated with the proper header labels: id denotes a subject identifier (e.g., *A101*), listnum denotes a unique list identifier per subject (e.g., *1* through *3* if a participant has three lists), and item denotes the participant responses (e.g., *dog, cat*, etc.). Responses within each list should be sorted in chronological order. Three other columns are optional: category denotes a fluency category label (e.g., *animals*), group is used to subset participants in the data based on meaningful experimental conditions or participant characteristics (e.g., *Monolinguals*), and rt denotes the inter-item response time for each response. The data file may also contain any number of additional columns, but these columns are ignored by SNAFU. Provided are some sample code snippets for importing data from the included fluency data (see Code Snippet 2).

**Code Snippet 2:**
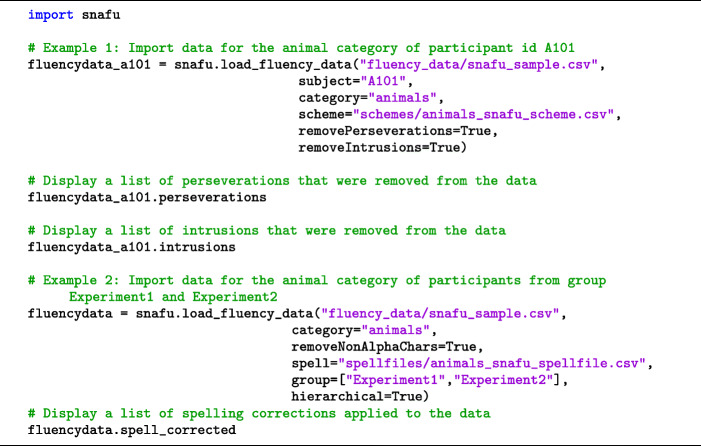
Loading fluency data into Python with SNAFU

The first argument to snafu.load_fluency_data is a string denoting the filename of the data. By default, SNAFU will load data from all participants, groups, and categories. You can filter the data before importing using the optional parameters subject, group, and category. In Code Snippet 2 (Example 1), only the responses for subject A101 in the animal category are imported, while in Code Snippet 2 (Example 2) animal fluency data from all participants in the Experiment 1 and Experiment2 groups are imported.

Non-alpha characters (including spaces) can be removed from responses by setting removeNonAlphaChars to *True* (default *False*). Known spelling errors can be automatically detected and corrected by optionally specifying a spell file. This file takes the form of a two-column CSV file in the format correct-spelling,incorrect-spelling (e.g., zebra,zebru). An extensive dictionary of over 2000 animal misspellings is provided in the GitHub repository, which is the collective effort of the authors and research assistants over the last three years. These spelling errors have been manually detected in previously collected fluency data of several hundred participants, spanning different ages and levels of education. If no spell file is specified, SNAFU will not make any spelling corrections. Perseverations (repetitions within a list) can be excluded by setting removePerseverations to *True* (default *False*). When toggled, verbatim perseverations are removed *after* spelling corrections are applied, if applicable.

Out-of-category or forbidden responses, known as intrusions, can be removed by setting removeIntrusions to *True* (default *False*). When toggled, a list of allowable responses must be provided by specifying a scheme file or a targetletter. A target letter (a single character) is intended for letter fluency tasks, whereas a scheme file is used for semantic fluency tasks. A scheme file is a two-column CSV file in the format category-label,member (e.g., Pets,dog). Responses not included as a member of at least one category in the scheme file are treated as intrusions. Scheme files are also used to compute cluster sizes and switches. Several animal category schemes are provided on GitHub. We include schemes from Troyer et al., (1997), an expanded categorization used by Hills et al., (2012), and further expansion of this categorization by the current authors and research assistants. The largest animal scheme file includes almost 900 animals, but custom scheme files can be used as well.

The fluency data can be imported in two ways. Setting hierarchical to *True* preserves the hierarchical structure, which groups multiple lists to a single participant. This is useful for calculating participant-level fluency statistics when each participant has completed multiple lists. When hierarchical is set to *False* (default), SNAFU treats each fluency list as if they came from a different participant. This option may be used to calculate list-level fluency statistics, which is useful when each participant has only one fluency list. This structure can also be toggled after the data are imported using the hierarchical and nonhierarchical methods. For example, in Example 2, the fluency data are imported into variable fluencydata hierarchically but can be switched to a non-hierarchical format using fluencydata.nonhierarchical().

After the data are loaded, fluencydata.labeledlists will contain the imported fluency lists. Alternatively, fluencydata.lists will return the same fluency lists in which each unique response is replaced with an integer. A data dictionary fluencydata.items specifies the mapping between the integer representation and string format for each participant. Lists are ordered first by participant (alphabetically) and then by list number (numerically). The ordering of lists is preserved in fluencydata.listnums, while the ordering of subjects is preserved in fluencydata.subs.

fluencydata.irts returns the inter-item response times for each fluency response. fluencydata.groupnumnodes returns the total number of unique responses across all participants. When the data are arranged hierarchically, fluencydata.numnodes specifies the number of unique responses for each participant.

### Fluency statistics

SNAFU can be used to calculate statistics on your fluency data. Here we provide a summary of how to compute common fluency statistics on a dataset.

#### Cluster sizes and switches

Verbal fluency data is typically clustered into sub-categories. For example, when listing animals a participant may list *bird, dog,* and *cat* (all from the category of pets) before switching to another cluster such as *zebra, lion,* and *hippo* (all zoo animals). Letter fluency data is often clustered by words that start with same few letters (e.g., *cart, can, cap* all begin with *ca*).^2^ Clustering and switching have been identified as two key components of memory search in verbal fluency tasks (Troyer et al., 1997). Although many measures summarizing clustered data have been proposed (Abwender et al., 2001), the most commonly used measures are average cluster size (number of responses per cluster) and total number of cluster switches in a list.

**Fig. 1:**
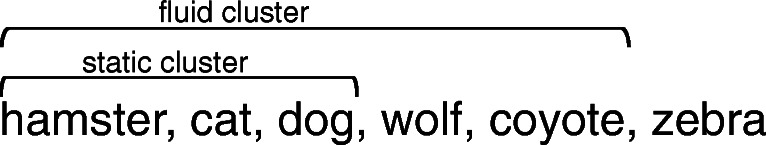
A static cluster begins at the termination of a previous cluster and ends when the next word does not share a category label with every item in the cluster. Above, *hamster, cat,* and *dog* are all pets, but *wolf* is not. A fluid cluster begins at the termination of a previous cluster and ends when the next word does not share a category label with the previous item only. Above, *dog* and *wolf* are both canines (no cluster switch), but *coyote* and *zebra* do not share a category label (cluster switch)

Cluster boundaries can be defined in at least two different ways (Hills et al., 2009; Hills et al., 2015). A fluid cluster switch occurs when the next word in a list does not share a category label with the previous word. A static cluster switch occurs whenever the next word does not share a category label with *any* of the previous words since the start of the last cluster. See Fig. 1.

SNAFU can compute the number of cluster switches and average cluster size in each list, for both semantic and letter fluency data (see Code Snippet 3). The first argument to snafu.clusterSwitch or snafu.clusterSize is a set of labeled fluency lists. The second argument specifies a clustering scheme to be used. For semantic fluency data, this is the name of a scheme file: a two-column csv file that specifies one or more category labels for each response in the format category-label,member (e.g., ZooAnimals,elephant). For letter fluency data, the argument may be an integer that specifies the number of consecutive letters to use as a category label. For example, 2 will use the first two letters of the word as its category label (e.g., *ca* for the response *cartoon*). Several scheme files for the animal category are provided, but custom scheme files can be used. Both functions support an optional argument clustertype to indicate the clustering method, set to either *fluid* (default) or *static*. Cluster switches and sizes will include perseverations and intrusions, unless they are removed when loading the data (see Section 3). When the data are formatted non-hierarchically, the mean cluster size and number of cluster switches for each list is returned. These values can be paired up with their relevant subject and list identifiers using fluencydata.listnums. When formatted hierarchically, SNAFU will return a single value for each participant (i.e., averaging across lists). These values can be paired up with their relevant subject identifiers using fluencydata.subs.

**Code Snippet 3:**
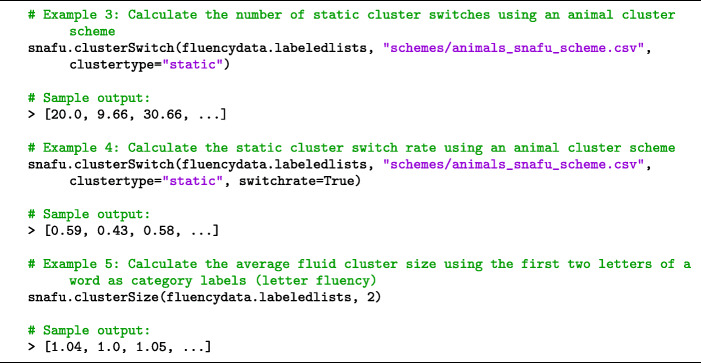
SNAFU calculates cluster switches and cluster sizes

Though the absolute number of cluster switches is commonly reported, this measure has been criticized because it is constrained by the total number of responses given by a participant (Hills et al., 2013). For example, an individual who generates 30 responses in a list can have up to 29 switches, whereas an individual who generates ten responses can have a maximum of nine switches. Consequently, though touted as a measure of cognitive switching ability (Troyer et al., 1997), the number of cluster switches is confounded with generative capacity. To mitigate this issue, we also implement a measure of switch rate (i.e., switches per item), which can be calculated by setting the optional parameter switchrate to *True* (default *False*) when using snafu.clusterSwitch.^3^

#### Perseverations

A perseveration occurs when a response is repeated twice within the same fluency list. Some perseverations can be easily identified, while other cases are more subjective. For example, different forms of the same word are typically disallowed (e.g., *eat* and *eating*) and treated as perseverations. Other cases are more ambiguous: if a participant lists *count* and *counter*, should the latter be treated as a perseveration because it is a form of *count* (i.e., one who counts), or should it be treated as an entirely new word (i.e., a kitchen counter)?

Many other ambiguous cases can arise. Suppose a partic ipant lists *cougar, catamount, mountain lion* and *puma* when listing animals. Scientifically, all of these names refer to the same species (*Puma concolor*). Should all responses after *cougar* be treated as perseverations? Some coding schemes specify that superordinate categories (e.g., *bird*) should not be counted when subordinate members of that category (e.g., *dove*) are also listed (Raoux et al., 2008). Some responses can be treated as either perseverations or intrusions depending on the experimenter’s interpretation. For example, if a participant lists *human* and *baby*, should the latter be treated as a perseveration because baby likely refers to a human of a specific age, or as an intrusion (Section 3) because *baby* is a generic term that does not refer to a specific animal?

Only responses that are repeated verbatim are treated as perseverations in SNAFU. However a customizable spell-check file allows detection of other perseverations by canonicalizing responses. The experimenter must manually specify which responses will be changed (e.g., spell-correcting *eating* to *eat*, or *baby* to *human*.) An extensive animal spell-check file with over 2000 fixes is provided on GitHub, including many canonicalizations.

Perseverations can be counted using snafu.perseverations. To return a list of the perseverations in each fluency list (rather than a count of perseverations), use snafu.perseverationsList. See Code Snippet 4. If the data are formatted non-hierarchically, snafu.perseverations returns the number of perseverations in each list. If formatted hierarchically, the function will return the average number of perseverations for each participant (across all lists).

**Code Snippet 4:**
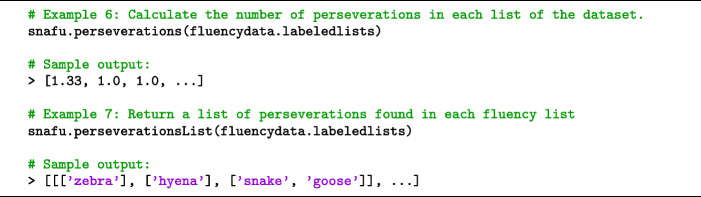
SNAFU can be used to detect and count perseverations

#### Intrusions

Responses that do not belong to the target category (e.g., animals) are called intrusions. While intrusions are rare for healthy participants, they are seen in older adults with clinical memory impairments (Tröster et al., 1989).

Identifying intrusions in letter fluency data is straightforward: if a response does not begin with the target letter, then it is an intrusion. Identifying intrusions in semantic fluency data can be subjective. For example, is a fictioal creature such as a *unicorn* an animal? What about single-celled *amoeba*, or generic labels such as *pet* or *mare*?

There is no standard for identifying intrusions, leading to inconsistency across the literature (Jordan, 2014). Moreover, researchers rarely provide a concrete set of criteria used to identify intrusions or a complete list of intrusions that were identified. SNAFU identifies intrusions using a customizable dictionary of allowable responses. We provide a list of allowable responses for the animal category (the cluster scheme file). We do not claim that this list is exhaustive, and will continue to update the list on GitHub over time.

To count intrusions, use snafu.intrusions. To return a list of the intrusions in each fluency list, use snafu.intrusionsList. See Code Snippet 5. If the data are formatted non-hierarchically, snafu.intrusions returns the number of intrusions in each list. If formatted hierarchically, the function will return the average number of intrusions for each individual (across all lists).

**Code Snippet 5:**
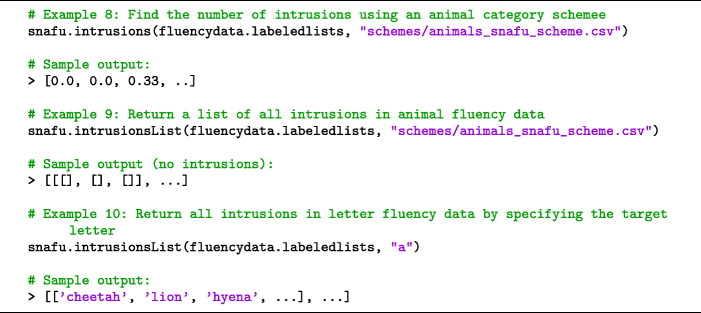
SNAFU can be used to detect and count intrusions

#### Word frequency, age of acquisition, and other word measures

Another measure used to score verbal fluency data is word frequency. SNAFU will calculate the average word frequency for all responses in a list (see Code Snippet 6). We include the SUBTLEXus database (Brysbaert and New, 2009), which lists the frequency of a word per million words in a large corpus of English subtitles.^4^ Researchers can provide their own database and optionally specify a value for words not included in the database with the missing parameter (e.g., the default for word frequency is *0.5*, as in Kuperman et al., (2012)). A custom database should be in the form of a CSV file and each line should contain one word in the format word,value; refer to the included age-of-acquisition or frequency databases as an example.

**Code Snippet 6:**
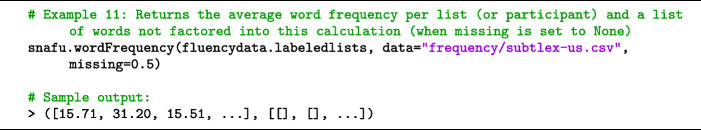
Calculating word frequency in SNAFU

SNAFU can also be used to calculate the average age-of-acquisition for responses (see Code Snippet 7). We provide the age-of-acquisition norms from Kuperman et al., (2012) as an example.^4^

**Code Snippet 7:**
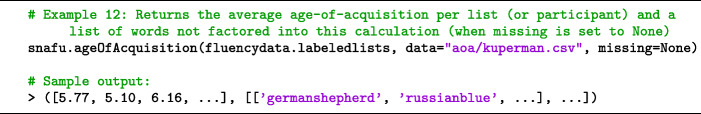
Calculating age-of-acquisition in SNAFU

This general approach is flexible enough to work with other word measures, such as ratings of abstractness or valence (see Code Snippet 8). By default, snafu.wordStat provides no data file (one must be specified) and words not in the data file are ignored (i.e., missing defaults to *None*).

**Code Snippet 8:**
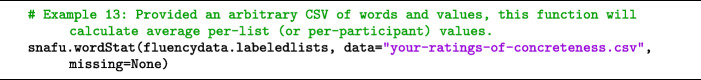
Calculating other word-level statistics in SNAFU

### Associative semantic networks

Traditionally, the analysis of fluency data has been limited to behavioral measures such as the number of items listed or the number of cluster switches. More recently, fluency data has been used to estimate latent semantic networks of groups and individuals (Zemla & Austerweil, 2018). A semantic network is a representation consisting of a set of nodes (one for each word), and a set of edges that connect nodes that are semantically similar (e.g., *horse* and *zebra* may be connected by an edge). These networks are one way to encode knowledge through the interconnection of concepts.

While several methods for estimating networks from fluency data exist, code or tools for constructing networks using these methods are not always publicly available or easy to use. Several large-scale semantic networks are publicly available, including semantic networks constructed from free association norms (De Deyne et al., 2019; Nelson et al., 2004). Other commonly used networks are derived from lexical databases such as WordNet (Griffiths et al., 2007; Miller, 1995). While these networks have provided significant value to language and memory research, there are limitations to using them. The participant sample and methodological choices used to construct these networks may bias these networks, and re-use of these networks may amplify these differences potentially leading to a biased literature. This is not a criticism of these networks in particular; it is commonly accepted that overreliance on a single subject pool may lead to conclusions that do not always generalize (e.g., Henrich et al., (2010)). Using diverse subject pools and methods for constructing networks allows researchers to provide converging evidence for specific psychological theories of interest.

One reason that researchers often rely on publicly available semantic networks is convenience. This convenience comes at a price: existing semantic networks place limitations on the analysis validity and kinds of analysis that are possible. Because these networks are constructed from many participants, it is not possible to assess individual or group differences in semantic memory. For example, a language researcher may be interested in comparing the semantic networks of monolinguals and bilinguals. Moreover, since the subject pool used to generate these networks is not available for subsequent testing, it is impossible to relate the semantic networks of individuals or groups to performance on other tasks (such as a working memory or intelligence test). In contrast, constructing and analyzing networks from fluency data can be tedious and require a significant time commitment. SNAFU mitigates this difficulty by implementing several network estimation methods.

Estimating representations from fluency data remains a challenging computational problem. For example, Voorspoels et al., (2014) found that the singular value decomposition procedure used by Sung et al., (2012) to estimate and compare semantic representations between typical and schizophrenic individuals was unreliable (though see Sung et al., (2016), for a rebuttal). Although the field has not converged upon a set of standard practices for estimating semantic networks, Zemla and Austerweil (2018) provide validation of several computational methods, suggestions for best practices, and a discussion of their limitations.

### Network estimation

SNAFU allows users to estimate a semantic network representation from fluency data. SNAFU provides several methods for estimating networks, briefly described in Table 1. Zemla and Austerweil (2018) discuss the computational details and psychological validity of each method and provide guidance on selecting a network estimation method and assessing its validity. Here, we limit our discussion to how to estimate networks using SNAFU. Fluency lists should be structured non-hierarchically to estimate a network, except for the hierarchical U-INVITE method. Some estimation methods allow for parameterization, though a default parameterization is applied. Network estimation examples are shown in Code Snippet 9, and additional details are provided in the accompanying demo files on GitHub.

**Table 1:** A description of each network estimation method

Network estimation method	Brief description
First Edge	The first two items in each fluency list are connected by an edge (Abrahao et al., 2013)
Naive Random Walk	All adjacent items in a fluency list are connected by an edge (Jun et al., 2015)
Pathfinder	The distance between each pair of items is measured and the union of all minimum spanning
	trees is preserved (Paulsen et al., 1996)
Correlation Based Network	The correlation between each pair of items is measured and pairs with the highest correlations
	are treated as edges (Kenett et al., 2013)
U-INVITE	The maximum likelihood network is estimated assuming data is generated from a censored
	random walk (Zemla and Austerweil, 2018)
Hierarchical U-INVITE	U-INVITE networks are estimated for each participant in addition to a latent group network
	(prior) (Zemla & Austerweil, 2018)

**Code Snippet 9:**
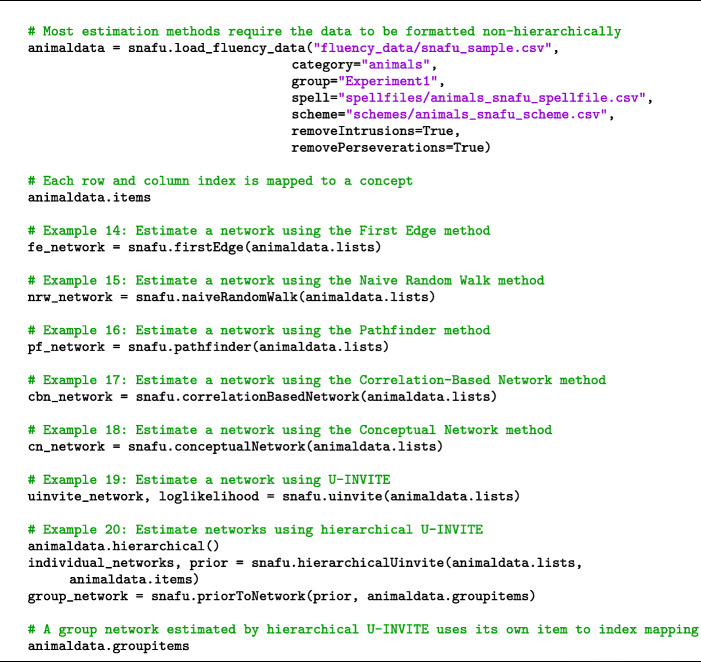
Estimating networks from fluency data in SNAFU

An estimated network is represented as a symmetrical matrix of zeroes and ones. Each row or column in the matrix represents a concept. A value of one in the matrix denotes an edge between two concepts, while zero denotes no edge.

U-INVITE networks pose some additional constraints. Perseverations are not allowed in the data (though see Zemla and Austerweil (2019), for a workaround).^5^ Hierarchical U-INVITE is used to simultaneously estimate an individual network for each participant as well as a group network for all participants. The method requires multiple fluency lists (3+) per individual, and can be very computationally intensive; for even moderately sized datasets, we recommend using parallelization (i.e., cluster computing).

Once a network has been estimated, you can write an edge list to a file (see Code Snippet 10).

**Code Snippet 10:**
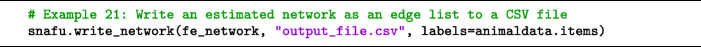
Exporting networks in SNAFU

### Graphical user interface (GUI)

**Fig. 2:**
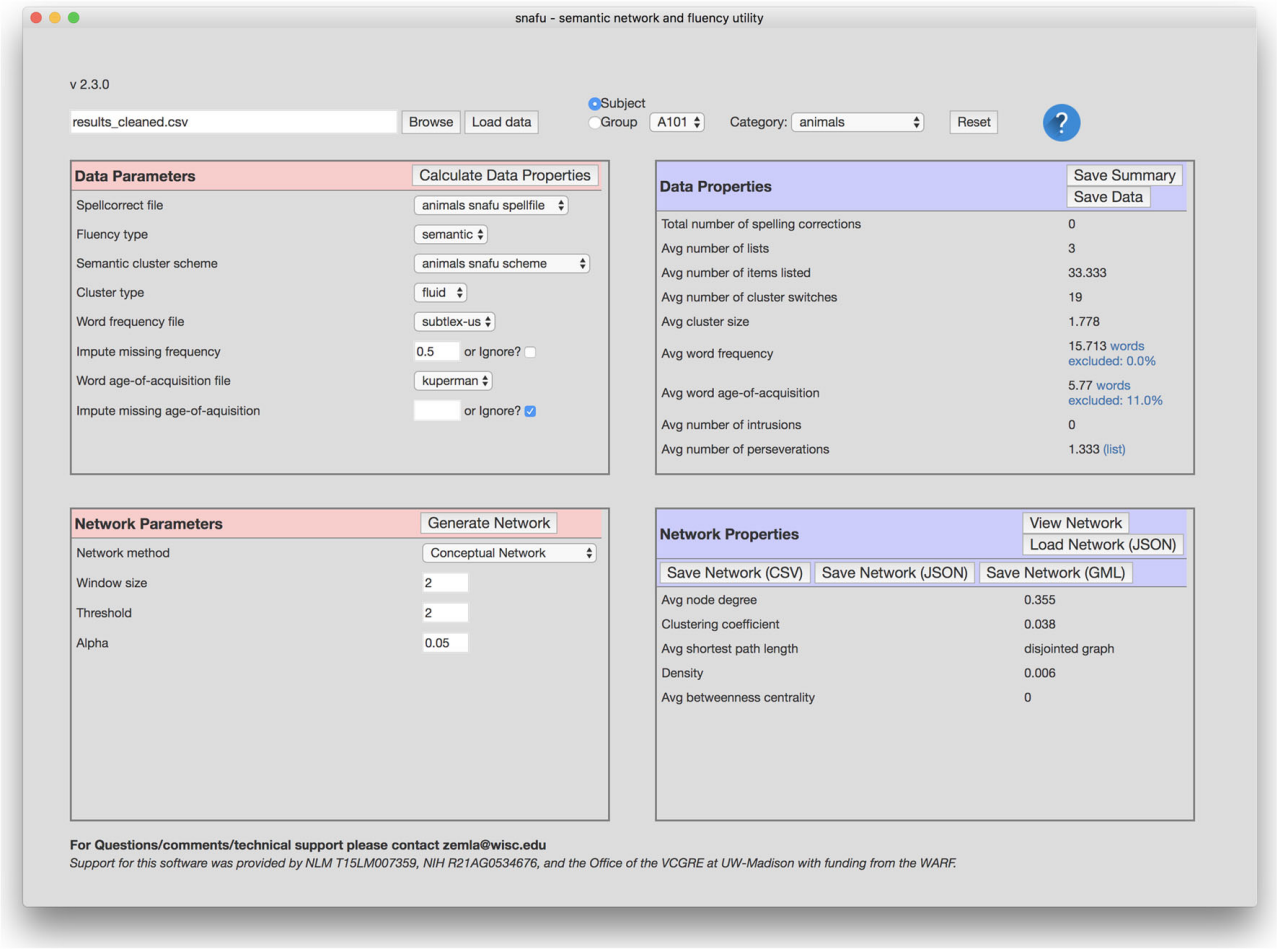
The GUI allows you to see at a glance many properties of the fluency, as well as properties of a network generated from the data

**Fig. 3:**
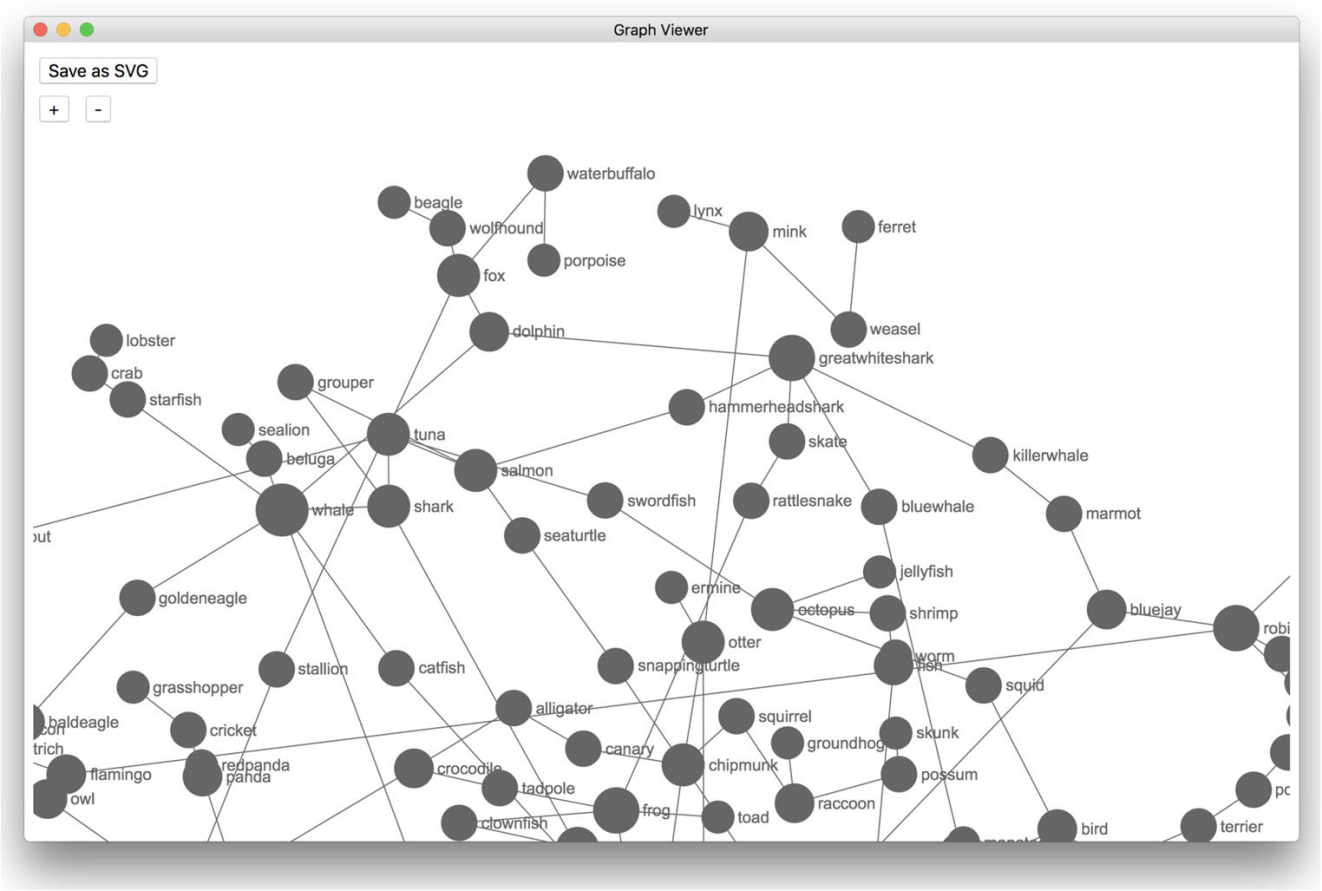
The GUI allows you to explore a network generated from the fluency data. You may click on a node to see its neighbors, zoom in and out, or drag nodes around

A graphical front-end to SNAFU is also available that does not require any programming experience (shown in Fig. 2). While its functionality is more limited than the Python library, it provides an easy way to compute and display many properties of the data with limited effort. Like the Python library, the GUI also allows you to generate statistics and networks from fluency data using a variety of methods. Additionally, the GUI includes a network viewer (shown in Fig. 3) that allows you to explore a visualization of the estimated network.

*Browse* is used to locate a fluency data file and *Load Data* is used to import the data. Once the data are loaded, you can *Calculate Data Properties* (fluency statistics) using the top-left box. A summary of the data are populated in the top-right box (*Data Properties*).

*Save Summary* is used to save a summary file in JavaScript Object Notation (JSON) format. The file contains both the summary data (shown in the *Data Properties* box) as well as the parameters used to generate that data (shown in the *Data Parameters* box). This file contains all of the details necessary to reproduce the analysis (except the data itself), and includes useful information such as: lists of spelling corrections, perseverations, intrusions, and other detailed information that is often omitted from manuscripts.

*Save Data* is used to export a CSV data file that contains list-level values for each fluency measure. This data file can be used for further analysis outside of SNAFU. *Generate Networks* in the bottom-left box can be used to estimate a network from the data. You can then *View* the network, export the network in several network formats, or import previously generated networks.

The software is available for both macOS and Windows, and can be downloaded from http://alab.psych.wisc.edu/snafu.

## Network estimation example

In this section, we provide a case study in using SNAFU to estimate a semantic network of foods.

### Participants and procedure

Fifty participants (ages 18–62; mean age 34.4; 42% female) located in the United States were recruited from Amazon’s Mechanical Turk. Participants completed three categories of the semantic fluency task (animals, tools, and foods). Each category was completed three times, for a total of nine lists. The order of the categories was pseudo-randomized such that each triplet of lists contained one of each category, and no category was repeated twice in a row. Participants had three minutes to complete each list. Responses were entered into a text box (one at a time), and each response faded from the screen (fade animation took 800 ms) to avoid memory cuing from previously entered responses.

We provide a network analysis of the food fluency data. We do not examine the tools or animals data in this manuscript. (A network analysis of the animal data was previously reported in Zemla and Austerweil (2018)). Below, we used the Conceptual Network method (Goñi et al., 2011) to construct a group-level semantic network.

### Results

Spelling errors were manually corrected and incorporated into the spelling dictionary, and synonymous items were canonicalized (e.g., *blueberries* to *blueberry*). Each list contained 35.6 responses on average, including an average of 0.29 perseverations per list (43 in total), which were not removed from the data prior to network analysis. The data also contained five non-foods or overly broad food categories.

**Fig. 4:**
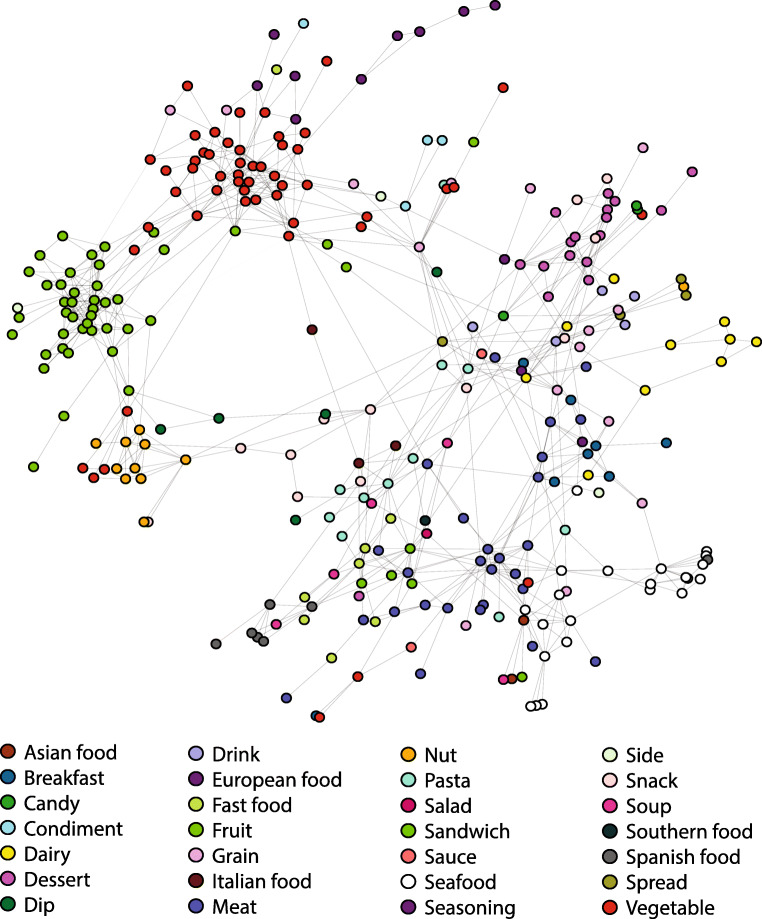
A network of foods generated using the Conceptual Network method. Nodes are color-coded to denote their primary category (determined a priori)

**Fig. 5:**
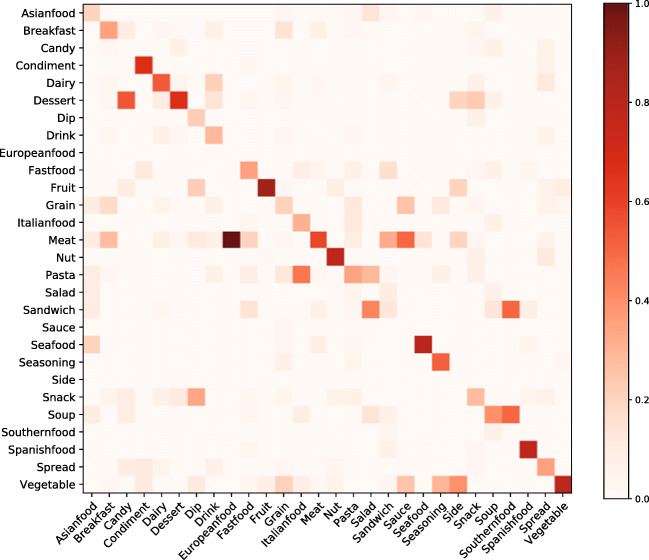
This heatmap shows the proportion of edges that go from a node of one category (*x*-axis) to another category (*y*-axis). As expected, a large majority of edges are intra-category, as indicated by the diagonal

The resultant network consisted of 337 disconnected components: one large connected component, and 336 smaller components of no more than two nodes each. Figure 4 shows the largest connected component of a network. This component consists of 298 nodes and 698 edges. It has a clustering coefficient of .32, an average node degree of 4.68, and an average shortest-path length of 4.84. Each node was assigned to one of 28 categories by a research assistant and one of the authors.^6^ Nodes in the network are colored according to their primary category. As expected, nodes that belong to the same category tend to be connected to other nodes of the same category. In total, 63.1% of edges are intra-category edges and the remaining edges are inter-category edges. The heatmap in Fig. 5 shows relations between categories computed by the number of inter- and intra-category edges.

## Fluency measure validation

We validate the more traditional fluency measures in SNAFU in two ways. First, we compare hand-coded calculations of fluency measures to SNAFU’s automated calculations on the same dataset. Second, we provide an example of how to use SNAFU to conduct typical fluency data analyses. Specifically, we show that fluency performance declines with age, and fluency performance declines in a population of individuals with mild cognitive impairment (MCI).

### Participants and methods

We obtained animal semantic fluency data collected by the Wisconsin Registry for Prevention of Alzheimer’s (WRAP; Johnson et al., (2018)). As part of WRAP, healthy and at-risk adult participants were recruited for a longitudinal study examining risk factors for Alzheimer’s disease (AD). Each participant completed an extensive neuropsychological battery of tests at each visit, which included a one minute version of the animal semantic fluency task. Participants returned four years after baseline for a second visit, and every two years thereafter. The animal fluency task was added to the WRAP battery in 2013 while the third wave of visits was underway, and was collected at all visits since. Our corpus consists of a convenient subset of those visits (i.e., a random sample that we have digitally transcribed).

Participants were an average of 63.9 years old at their first fluency visit (range, 39–80) and 70% of participants were female. Participants completed an average of 15.7 years of education (range 10–20^7^). At each visit, participants were classified as cognitively unimpaired, amnestic MCI (aMCI), non-amnestic MCI (naMCI), or both aMCI and naMCI. 15.3% of visits were from participants who were classified as having aMCI, while 6.6% were classified as having naMCI (2.3% were classified as both). The remaining visits were classified as cognitively unimpaired.

aMCI classification at each visit was based on an algorithmic flagging procedure developed by Koscik et al., (2014). Participants completed a battery of neuropsychological tests and questionnaires (Koscik et al., 2016; Sager et al., 2005), and a factor analysis was performed on the data. Of the six factors that were identified (Dowling et al., 2010), two factors (derived from the Rey Auditory-Verbal Learning Test; Lezak et al., (2004)) were used for classification of aMCI: Immediate Memory (IM) and Verbal Learning & Memory (VLM). Robust norms were developed using a population of individuals without a family history of AD (Koscik et al., 2014). Individuals were classified as aMCI if they scored more than 1.5 standard deviations below predicted scores on both IM and VLM based on age, gender, and Wide Range Achievement Test-III decile (Wilkinson, 1993). An identical procedure was used to classify naMCI using the Working Memory and Speed & Flexibility factors. This procedure differs from the consensus panel approach to classifying MCI (Albert et al., 2011) and was developed in part to identify cognitive declining individuals in a population of middle-aged (not elderly) and highly educated participants such as WRAP. The procedure is more liberal than clinical diagnosis, and many of the participants in our sample classified as aMCI are pre-clinical MCI.

We examined 1066 fluency lists generated by 796 participants (1.34 lists per participant, range 1–3). Fluency data was coded by five human raters for number of cluster switches, average cluster size, number of perseverations, number of intrusions, and number of unique valid responses. Twenty percent of all lists were coded by two separate raters. There was high agreement between raters for all fluency measures (Cronbach’s *α* > .9). The dataset we analyze partially overlaps with data previously reported (Mueller et al., 2015). This dataset was collected and coded independently of SNAFU’s development. For the following analyses, we auto-corrected any spelling mistakes using SNAFU’s provided list of spelling errors. SNAFU clusters were demarcated using the animal clusters provided in SNAFU. We did not modify these files for the present dataset. In the analyses below, we evaluate how well fluency measures computed by SNAFU (“SNAFU-coded”) align with the same measures computed by human coders (“hand-coded”).

### Results

#### Number of responses

The most commonly reported fluency measure is the number of responses given by a participant. Here, we report the total number of responses given, including perseverations and intrusions. Fifty-nine lists (5.5%) were coded by SNAFU as having a different number of responses; the majority, 35 of these 59 lists (3.3% of the total), were off by one item. Overall, there is high agreement for total number responses, *r*(1062) = 0.98, *RMSE* = 0.99. (Two outliers were excluded because the hand-coded total number of responses fell more than three standard deviations from the mean number of hand-coded number of responses.)

#### Perseverations

Hand-coded perseverations correlated highly with those detected by SNAFU, *r*(1040) = 0.8, *RMSE* = 0.48, excluding 24 outliers whose hand-coded values were more than three standard deviations from the mean. Table 2 provides a confusion matrix showing the number of perseverations detected in each list. On average, SNAFU tends to overcount animal perseverations and produce few undercounts. The reason for this is that the default animal spell-correct file includes many canonicalizations that treat similar responses as identical. For instance, *African elephant* and *Asian elephant* are both canonicalized to *elephant*. This can be avoided by using a custom spell-correct file.

**Table 2:** The number of perseverations in each list, as coded by human raters (*x*-axis) and SNAFU (*y*-axis)

		Hand-coded perseverations							
		0	1	2	3	4	5	6	7
SNAFU coded	0	648	6	0	0	0	0	0	0
perseverations	1	70	200	2	0	0	0	0	0
	2	9	32	62	0	1	0	0	0
	3	1	1	8	12	0	0	0	0
	4	0	0	2	3	3	1	0	0
	5	0	0	0	1	1	0	0	0
	6	0	0	0	0	0	1	0	0
	7	0	0	0	0	0	0	0	1
	8	0	0	0	0	0	0	0	0
	9	0	1	0	0	0	0	0	0

#### Clustering

**Fig. 6:**
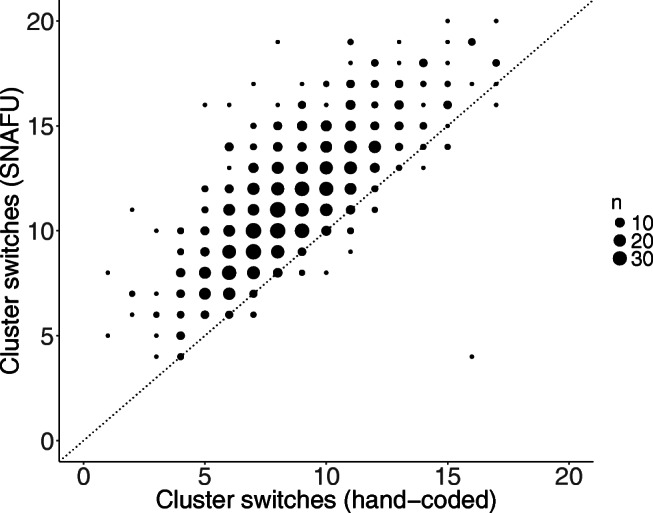
Shown is the number of cluster switches calculated by human raters (*x*-axis) and SNAFU (*y*-axis) for each list. The size of each *point* denotes the number of lists with that value. The *dotted line* represents the identity line

**Fig. 7:**
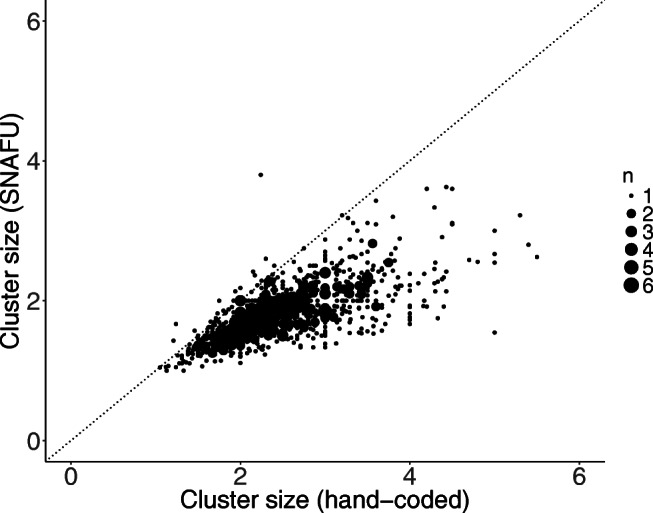
Shown is the average cluster size calculated by human raters (*x*-axis) and SNAFU (*y*-axis) for each list. The size of each *point* denotes the number of lists with that value. The *dotted line* represents the identity line

We computed static cluster switches in SNAFU and found they correlated highly with hand-coded cluster switches, *r*(1057) = 0.77, *RMSE* = 3.48, excluding six outliers whose hand-coded values were more than three standard deviations from the mean and one participant whose cluster switch score was missing. Similarly, static cluster sizes coded manually and by SNAFU were correlated highly, *r*(1055) = 0.70, *RMSE* = 0.85, excluding seven outliers and two missing values. Figures 6 and 7 show a comparison between human raters and SNAFU. Although these measures are highly correlated, SNAFU codes for more cluster switches (and smaller cluster sizes) compared to human coders. One possible explanation for this is that SNAFU uses a more granular concept space to demarcate clusters, whereas human coders might use a smaller set of broader categories.

#### Intrusions

We found that SNAFU’s count of intrusions had low agreement with a hand count of intrusions, *r*(1039) = 0.01, *p* = .65, *RMSE* = 0.79, excluding 29 outliers.

A closer look at the intrusions detected by SNAFU may explain why agreement is so low. In total, 298 intrusions were detected by SNAFU (249 unique), which is a small percentage of the 28,822 total responses in the dataset (709 unique). The vast majority of responses were accurately identified as non-intrusions. However the list of intrusions detected by SNAFU were mixed: while some are likely legitimate intrusions (*beers, elf, airplane, schoolbook*), many others were spelling errors that were not in the SNAFU spell-check file (*gazebra, gpig, nadger*), and a few valid animals that have not been categorized (*turkey vulture, butterfly fish, Honduran milk snake*) were classified as intrusions. Many other intrusions referred to non-specific animal terms or higher-order classifications (*mongrel, mammal, crustaceans, bacteria*) which may or may not have been coded as intrusions by the human raters. Because intrusions were very rare in general (approximately 1.5% of responses), small deviations may have a large impact on the correlation.

This mismatch highlights an important fact: though SNAFU is able to assist with automation of fluency scoring, it is important not to rely on its analysis without a closer inspection of the data. We recommend that the researcher examine the list of detected intrusions (using snafu.intrusionsList), add any misspellings to the dictionary and categorize any valid animals, and then re-run the analysis. Though SNAFU is unable to automate this process, it can greatly reduce the time required for human coders, as it is not necessary to manually sift through each fluency list.

#### Analysis example: Aging and mild cognitive impairment

Performance on the semantic fluency task is impaired in older adults (Troyer et al., 1997) and in individuals with Mild Cognitive Impairment (MCI; Nutter-Upham et al., (2008)). Using the same WRAP cohort as above, we examined how the fluency performance of a cohort of individuals changes with age and aMCI classification.

We fit a mixed-effect linear regression model for each of five dependent variables (total responses, cluster switches, average cluster size, intrusions, and perseverations) using either the SNAFU coding or hand-coding (i.e., ten models in total). We also fit two additional models treating word frequency and age-of-acquisition as dependent variables. Fixed effects for each model were age, aMCI status (binary), and their interaction. Participant was included as a random effect. We used the same outlier criteria as above for each model, excluding participants with missing data or values more than three standard deviations from the mean of the hand-coded data.

We found that overall, using hand-coded data or SNAFU produced similar results. For statistical comparisons, refer to Table 3. For visualization, we also include bar plots that separate “younger” and “older” adults using a median split (63.78 years). Overall, the data represent 903 cognitively unimpaired visits (473 younger, 430 older) and 163 aMCI visits (74 younger, 89 older).

**Table 3:** Comparison of SNAFU and hand-coded data

	Hand-coded						SNAFU coded					
	Age		aMCI		Age × aMCI		Age		aMCI		Age × aMCI	
	*t*	*p*	*t*	*p*	*t*	*p*	*t*	*p*	*t*	*p*	*t*	*p*
Num responses	− 2.08	.038^∗^	1.80	.073^‡^	− 2.03	.043^∗^	− 1.85	.065^‡^	1.87	.061^‡^	− 2.06	.040^∗^
Cluster switches	− 4.19	< .001^∗^	− 0.13	.90	0.10	.92	− 3.76	< .001^∗^	1.48	.14	− 1.67	.095^‡^
Cluster size	1.90	.056^‡^	0.58	.56	− 0.78	.43	1.21	.23	− 0.73	.46	0.65	.52
Intrusions	1.27	.21	− 0.19	.85	.21	.84	2.16	.031^∗^	− 1.57	.12	1.67	.097^‡^
Perseverations	0.73	.47	2.41	.016^∗^	− 2.41	.017^∗^	1.80	.073^‡^	2.25	.025^∗^	− 2.35	.019^∗^
Word frequency							2.01	.044^∗^	0.76	.45	− 0.68	.50
Age of acquisition							2.16	.031^∗^	− 1.29	.20	1.20	.23

^*^ p <.05; ^‡^*p* < .1

**Fig. 8:**
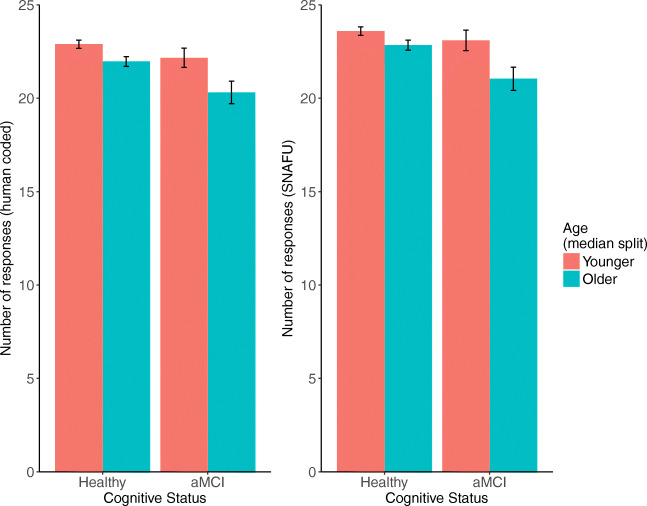
The number of responses calculated by hand (*left*) or SNAFU (*right*), separated by age (median split) and aMCI status

We found that older adults listed fewer responses, as did individuals classified with aMCI. In addition, we found an interaction such that individuals with aMCI declined more with age than cognitively unimpaired individuals. All results were significant (*p* < .05) or marginal (*p* < .1) using both SNAFU and hand-coded data. See Fig. 8.

**Fig. 9:**
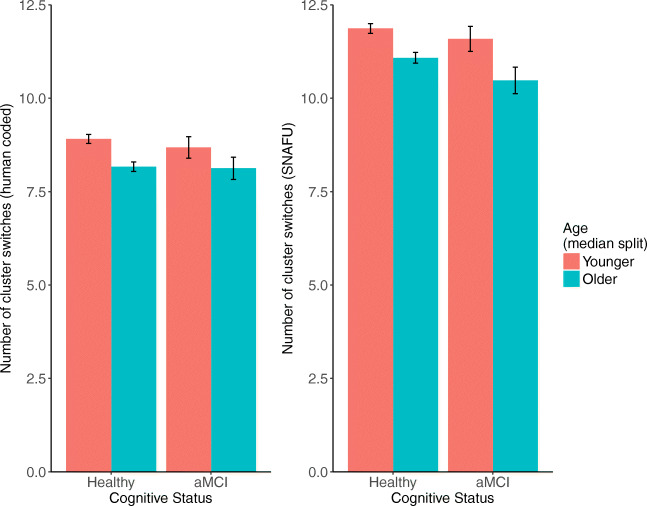
The average number of cluster switches calculated by hand (*left*) or SNAFU (*right*), separated by age (median split) and aMCI status

Participants switched clusters significantly less with age, assessed using both SNAFU and hand-coded data. Using SNAFU (but not the hand-coded data), we also found a marginal interaction where cluster switching was more impaired in older adults for the aMCI group than the unimpaired group. See Fig. 9.

**Fig. 10:**
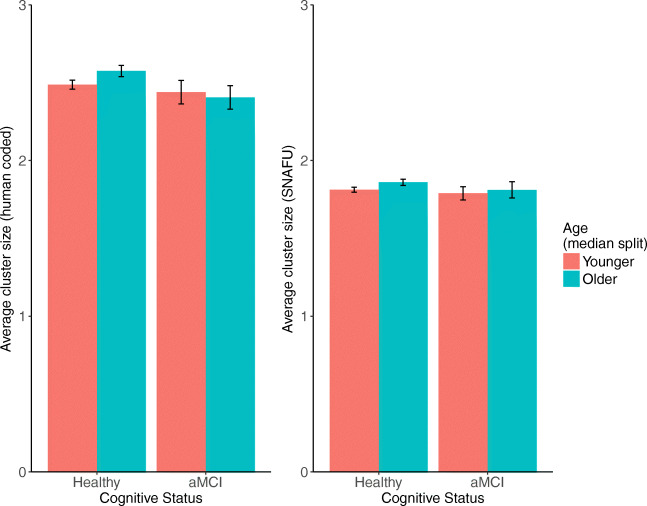
The average cluster size calculated by hand (*left*) or SNAFU (*right*), separated by age (median split) and aMCI status

Using the hand-coded data, we found that older adults produced marginally larger cluster sizes than younger adults. This effect was not replicated with SNAFU. See Fig. 10.

**Fig. 11:**
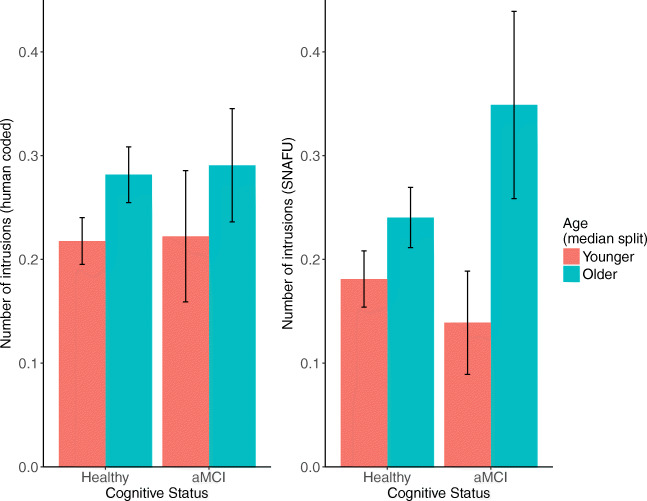
Intrusions calculated by hand (*left*) or SNAFU (*right*), separated by age (median split) and aMCI status

We found that using the SNAFU coding (but not the hand-coded data), older adults generated significantly more intrusions, as well as a marginal interaction with aMCI status: the difference in number of intrusions between older and younger adults is larger in the aMCI group. We hesitate to strongly endorse this finding for two reasons. First, intrusions were uncommon in the dataset overall. Second, many of the animal intrusions identified by SNAFU are ambiguous. One possibility is that these results are driven by an increase in use of generic terms by older and impaired individuals (e.g., *mammal, mongrel*) that are coded as intrusions by default in SNAFU. See Fig. 11.

**Fig. 12:**
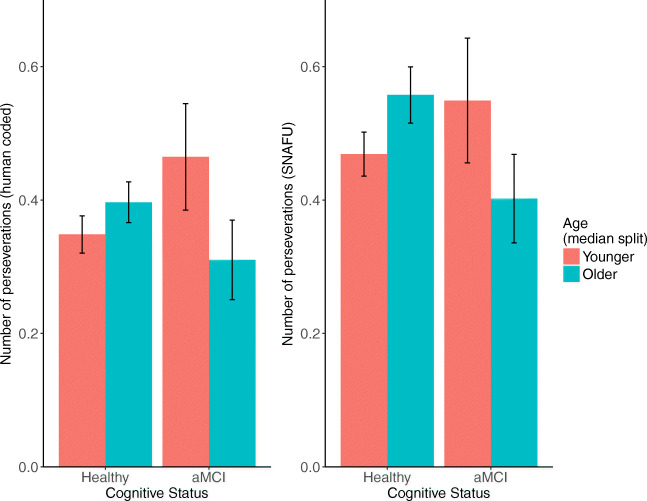
Number of perseverations calculated by hand (*left*) or SNAFU (*right*), separated by age (median split) and aMCI status

With both hand-coding and SNAFU, aMCI participants generated significantly more perseverations. However, this was qualified by an unexpected interaction: among unimpaired individuals older adults tended to generate more perseverations, whereas among aMCI individuals younger adults tended to generate more perseverations. Using SNAFU coding (but not hand-coding), there was a marginal effect of age where older adults generated more perseverations. See Fig. 12.

**Fig. 13:**
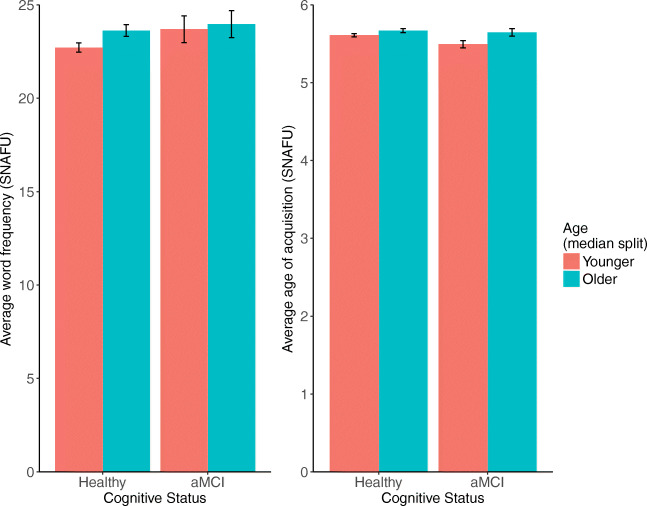
Average word frequency (*left*) and age-of-acquisition (*right*) calculated by SNAFU, separated by age (median split) and aMCI status

Average word frequency and average age-of-acquisition were calculated using SNAFU only. For both variables, we found a significant main effect such that older adults tended to list animals that were higher in frequency and higher in average age-of-acquisition. See Fig. 13.

## Discussion

Although verbal fluency tasks are widely prevalent in psychology and other domains, no standardized and publicly accessible tool exists for analyzing fluency data and constructing networks from fluency data. SNAFU automates verbal fluency data analysis, minimizing the time needed to perform tedious analysis tasks. SNAFU allows readers to easily reproduce and verify important statistics from fluency data. Furthermore, the default settings in SNAFU, derived from common practices in the literature, are chosen to encourage standardization across the fluency literature. It is our hope that SNAFU will improve validity, reproducibility, and standardization across the verbal fluency literature.

## Footnotes

1 pip can be installed via https://pip.pypa.io/

2 Manually coded letter fluency clusters sometimes include homonyms or rhymes (Troyer, 2000). SNAFU does not currently recognize these clusters.

3 Switch rate is also problematic as a measure of cognitive switching because it is mathematically very similar to (and highly correlated with) cluster size. We encourage researchers to take steps to disentangle these measures when assessing switching ability (e.g., by examining the partial correlation between cluster switches and a relevant dependent variable while controlling for the total number of responses).

4 This dataset was not compiled by the authors and should be cited separately when used in analysis.

5 The demo file *fit_uinvite_network.py*, included in the GitHub repository, describes how to implement this solution

6 Although the authors were not blind to the experiment’s purpose, the foods were sorted into categories prior to network estimation.

7 Education years were truncated at 20 for anyone reporting more than 20 years.

## References

[CR1] Abbott J, Austerweil J, Griffiths T (2015). Random walks on semantic networks can resemble optimal foraging. Psychological Review.

[CR2] Abrahao, B., Chierichetti, F., Kleinberg, R., & Panconesi, A. (2013). Trace complexity of network inference. In: Proceedings of the 19th ACM SIGKDD international conference on knowledge discovery and data mining, ACM, pp. 491–499.

[CR3] Abwender DA, Swan JG, Bowerman JT, Connolly SW (2001). Qualitative analysis of verbal fluency output: Review and comparison of several scoring methods. Assessment.

[CR4] Albert MS, DeKosky ST, Dickson D, Dubois B, Feldman HH, Fox NC, Gamst A, Holtzman DM, Jagust WJ, Petersen RC (2011). The diagnosis of mild cognitive impairment due to Alzheimer’s disease: Recommendations from the National Institute on Aging–Alzheimer’s Association workgroups on diagnostic guidelines for Alzheimer’s disease. Alzheimer’s & Dementia.

[CR5] Bousfield WA, Sedgewick CHW (1944). An analysis of sequences of restricted associative responses. The Journal of General Psychology.

[CR6] Brysbaert M, New B (2009). Moving beyond Kučera and Francis: A critical evaluation of current word frequency norms and the introduction of a new and improved word frequency measure for American English. Behavior Research Methods.

[CR7] Collins AM, Quillian MR (1969). Retrieval time from semantic memory. Journal of Verbal Learning and Verbal Behavior.

[CR8] De Deyne S, Navarro DJ, Perfors A, Brysbaert M, Storms G (2019). The “small world of words” English word association norms for over 12,000 cue words. Behavior Research Methods.

[CR9] Dowling NM, Hermann B, La Rue A, Sager MA (2010). Latent structure and factorial invariance of a neuropsychological test battery for the study of preclinical Alzheimer’s disease. Neuropsychology.

[CR10] Gollan TH, Montoya RI, Werner GA (2002). Semantic and letter fluency in Spanish–English bilinguals. Neuropsychology.

[CR11] Goñi J, Arrondo G, Sepulcre J, Martincorena I, de Mendizábal NV, Corominas-Murtra B, Bejarano B, Ardanza-Trevijano S, Peraita H, Wall DP (2011). The semantic organization of the animal category: Evidence from semantic verbal fluency and network theory. Cognitive Processing.

[CR12] Griffiths TL, Steyvers M, Tenenbaum JB (2007). Topics in semantic representation. Psychological Review.

[CR13] Helm-Estabrooks, N. (2001). Cognitive linguistic quick test: CLQT. PsychCorp.

[CR14] Henrich J, Heine SJ, Norenzayan A (2010). Most people are not weird. Nature.

[CR15] Hills, T.T., Jones, M.N., & Todd, P.M. (2009). Optimal foraging in semantic memory. In: Proceedings of the annual meeting of the Cognitive Science Society, pp 620–625.

[CR16] Hills TT, Jones MN, Todd PM (2012). Optimal foraging in semantic memory. Psychological Review.

[CR17] Hills TT, Mata R, Wilke A, Samanez-Larkin GR (2013). Mechanisms of age-related decline in memory search across the adult life span. Developmental Psychology.

[CR18] Hills TT, Todd PM, Jones MN (2015). Foraging in semantic fields: How we search through memory. Topics in Cognitive Science.

[CR19] Johnson SC, Koscik RL, Jonaitis EM, Clark LR, Mueller KD, Berman SE, Bendlin BB, Engelman CD, Okonkwo OC, Hogan KJ (2018). The Wisconsin Registry for Alzheimer’s Prevention: A review of findings and current directions. Alzheimer’s & Dementia: Diagnosis. Assessment & Disease Monitoring.

[CR20] Jordan, L.M. (2014). Verbal fluency: Norms for the Lakota population in semantic and phonemic fluency tasks. Masters Thesis.

[CR21] Jun, K.S., Zhu, X., Rogers, T.T., Yang, Z., & et al (2015). Human memory search as initial-visit emitting random walk. In: Advances in neural information processing systems, pp 1072–1080.

[CR22] Kenett YN, Wechsler-Kashi D, Kenett DY, Schwartz RG, Ben Jacob E, Faust M (2013). Semantic organization in children with cochlear implants: Computational analysis of verbal fluency. Frontiers in Psychology.

[CR23] Kim N, Kim JH, Wolters MK, MacPherson SE, Park JC (2019). Automatic scoring of semantic fluency. Frontiers in Psychology.

[CR24] Koscik RL, La Rue A, Jonaitis EM, Okonkwo OC, Johnson SC, Bendlin BB, Hermann BP, Sager MA (2014). Emergence of mild cognitive impairment in late middle-aged adults in the Wisconsin Registry for Alzheimer’s Prevention. Dementia and Geriatric Cognitive Disorders.

[CR25] Koscik RL, Berman SE, Clark LR, Mueller KD, Okonkwo OC, Gleason CE, Hermann BP, Sager MA, Johnson SC (2016). Intraindividual cognitive variability in middle age predicts cognitive impairment 8–10 years later: results from the Wisconsin Registry for Alzheimer’s Prevention. Journal of the International Neuropsychological Society.

[CR26] Kuperman V, Stadthagen-Gonzalez H, Brysbaert M (2012). Age-of-acquisition ratings for 30,000 English words. Behavior Research Methods.

[CR27] Lezak MD, Howieson DB, Loring DW, Fischer JS (2004). Neuropsychological assessment.

[CR28] Linz, N., Tröger, J, Alexandersson, J., & Konig, A. (2017). Using neural word embeddings in the analysis of the clinical semantic verbal fluency task. In *IWCS 2017 - 12th international conference on computational semantics, Montpellier, France* (pp. 1–7).

[CR29] Mikolov, T., Sutskever, I., Chen, K., Corrado, G.S., & Dean, J. (2013). Distributed representations of words and phrases and their compositionality. In: Advances in neural information processing systems, pp 3111–3119.

[CR30] Miller GA (1995). WordNet: A lexical database for English. Communications of the ACM.

[CR31] Monsch AU, Bondi MW, Butters N, Salmon DP, Katzman R, Thal LJ (1992). Comparisons of verbal fluency tasks in the detection of dementia of the Alzheimer type. Archives of Neurology.

[CR32] Mueller KD, Koscik RL, LaRue A, Clark LR, Hermann B, Johnson SC, Sager MA (2015). Verbal fluency and early memory decline: Results from the Wisconsin Registry for Alzheimer’s Prevention. Archives of Clinical Neuropsychology.

[CR33] Nasreddine ZS, Phillips NA, Bédirian V, Charbonneau S, Whitehead V, Collin I, Cummings JL, Chertkow H (2005). The Montreal Cognitive Assessment, MOCA: A brief screening tool for mild cognitive impairment. Journal of the American Geriatrics Society.

[CR34] Nelson DL, McEvoy CL, Schreiber TA (2004). The University of South Florida: Free association, rhyme, and word fragment norms. Behavior Research Methods, Instruments, & Computers.

[CR35] Nutter-Upham KE, Saykin AJ, Rabin LA, Roth RM, Wishart HA, Pare N, Flashman LA (2008). Verbal fluency performance in amnestic MCI and older adults with cognitive complaints. Archives of Clinical Neuropsychology.

[CR36] Open Science Collaboration (2015). Estimating the reproducibility of psychological science. Science.

[CR37] Paulsen JS, Romero R, Chan A, Davis AV, Heaton RK, Jeste DV (1996). Impairment of the semantic network in schizophrenia. Psychiatry Research.

[CR38] Quillian MR (1967). Word concepts: A theory and simulation of some basic semantic capabilities. Behavioral Science.

[CR39] Raoux N, Amieva H, Le Goff M, Auriacombe S, Carcaillon L, Letenneur L, Dartigues JF (2008). Clustering and switching processes in semantic verbal fluency in the course of Alzheimer’s disease subjects: Results from the PAQUID Longitudinal Study. Cortex.

[CR40] Ross TP, Calhoun E, Cox T, Wenner C, Kono W, Pleasant M (2007). The reliability and validity of qualitative scores for the controlled oral word association test. Archives of Clinical Neuropsychology.

[CR41] Sager MA, Hermann B, La Rue A (2005). Middle-aged children of persons with Alzheimer’s disease: APOE genotypes and cognitive function in the Wisconsin Registry for Alzheimer’s Prevention. Journal of Geriatric Psychiatry and Neurology.

[CR42] Simmons JP, Nelson LD, Simonsohn U (2011). False-positive psychology: Undisclosed flexibility in data collection and analysis allows presenting anything as significant. Psychological Science.

[CR43] Sung K, Gordon B, Vannorsdall TD, Ledoux K, Pickett EJ, Pearlson GD, Schretlen DJ (2012). Semantic clustering of category fluency in schizophrenia examined with singular value decomposition. Journal of the International Neuropsychological Society.

[CR44] Sung K, Gordon B, Schretlen DJ (2016). Semantic structure can be inferred from category fluency tasks via clustering analyses: Reply to Voorspoels others.(2014). Cortex; A journal devoted to the study of the nervous system and behavior.

[CR45] Tröster AI, Salmon DP, McCullough D, Butters N (1989). A comparison of the category fluency deficits associated with Alzheimer’s and Huntington’s disease. Brain and Language.

[CR46] Troyer AK (2000). Normative data for clustering and switching on verbal fluency tasks. Journal of Clinical and Experimental Neuropsychology.

[CR47] Troyer AK, Moscovitch M, Winocur G (1997). Clustering and switching as two components of verbal fluency: Evidence from younger and older healthy adults. Neuropsychology.

[CR48] Tulving, E. (1972). Episodic and semantic memory. In E. Tulving, & W. Donaldson (Eds.) *Organization of Memory* (pp. 382–402). New York: Academic Press.

[CR49] Voorspoels W, Storms G, Longenecker J, Verheyen S, Weinberger DR, Elvevåg B (2014). Deriving semantic structure from category fluency: Clustering techniques and their pitfalls. Cortex.

[CR50] Wilkinson GS (1993). Wide range achievement test–revision 3.

[CR51] Woods DL, Wyma JM, Herron TJ, Yund EW (2016). Computerized analysis of verbal fluency: Normative data and the effects of repeated testing, simulated malingering, and traumatic brain injury. PloS one.

[CR52] Zemla JC, Austerweil JL (2018). Estimating semantic networks of groups and individuals from fluency data. Computational Brain & Behavior.

[CR53] Zemla, J.C., & Austerweil, J.L. (2019). Analyzing knowledge retrieval impairments associated with Alzheimer’s disease using network analyses. Complexity 2019. 10.1155/2019/4203158 PMC6656530 31341377

